# “It’s about being healthy”; a novel approach to the socio-ecological model using family perspectives within the Latinx community

**DOI:** 10.1186/s12889-023-15005-2

**Published:** 2023-01-11

**Authors:** Bethany Korom, Meghan Malloy, Caroline Remmers, Mari Cevilla, Kelly Dione, Paula Papanek, Jeff Condit, David Nelson

**Affiliations:** 1 Medical College of Wisconsin, WI Milwaukee, USA; 2 United Community Center, Milwaukee, WI USA; 3 Program in Exercise Science, Marquette University, Milwaukee, WI USA; 4 Institute for Clinical and Translational Research, University of Wisconsin-Madison, WI Madison, USA

**Keywords:** Hispanic, Community-based intervention, Dynamic model, Family cohesion, Nutrition & physical activity

## Abstract

**Background:**

The Latinx community is at risk for obesity, type 2 diabetes, and other chronic illnesses. Culturally appropriate, community facing physical activity (P.A.) and nutrition programs may provide the basis for families to improve their health status. Our objectives are as follows: 1. To investigate synergistic factors within this type of program that play a role in creating an environment for participants to learn and practice healthy behaviors. 2. To apply factors into a novel model of components that support health and wellness. 3. To design an intervention for future implementation and evaluation.

**Methods:**

A two-year P.A. and nutrition program, *Families Inspired Together 4 Youth Empowered to Succeed* (FIT 4 YES), took place in Milwaukee, WI. with Hispanic families from 2018 to 2020 through a community-academic partnership. A pair of interviewers spoke with families who provided insight into the impact of the program. A grounded theory qualitative approach to code the transcripts guided the team to identify overarching themes.

**Results:**

Twenty-four interviews were conducted. Common themes indicated that children had a stronger belief in their abilities and confidence in peer support. Parents noticed their children increasing self-directed healthy behaviors. All families grew in their implementation of health and wellness.

**Conclusions:**

Three main components of FIT 4 YES contributed to its success: opportunities for engagement, supportive relationships, and the interplay of components that emerged from the interviews. Effective programs could include these components to make their outcomes more cohesive within the family. A novel model emerged that builds on the social-ecological model that emphasizes the dynamic interactions between these main components. Additional research is needed to evaluate the long-term effects and response by the community.

**Supplementary Information:**

The online version contains supplementary material available at 10.1186/s12889-023-15005-2.

## Background

According to the Centers for Disease Control and Prevention (2020), only 24% of children aged 6 to 17 are physically active 60 min per day. The prevalence of obesity among U.S. youth currently resides at 17.8% and severe obesity at 5.8%. Hispanic youth experience the highest obesity rates of 23.6% as compared to non-Hispanic white youth at 14.7% [1]. Multiple factors contribute to the decrease in physical activity and rise of childhood obesity that coincide with a social-ecological model (SEM) of community [2]. The SEM is a widely adapted model that demonstrates the interplay between individual, interpersonal, community and societal levels to conceptualize the numerous factors that influence one’s health. This model encourages interventions to target multiple levels rather than individual behaviors alone [3]. Though many interventions exist to support health and well-being, more meaningful intersections are needed across this social-ecologic framework [4, 5]. However, a critical barrier to progress exists: many intervention models are created without input from the families, participants, and communities about what may be most effective in working with community partnerships [6].

Several barriers across the SEM limit a program’s effectiveness and are important to address for the best chance at success [7]. At the individual level, inconsistent culture and language considerations may make participants feel like outsiders within program [8]. Multilingual Latinx families may benefit from bilingual mentors and peers in health-promoting programs, creating a culture of inherent inclusivity [9]. Improving intrapersonal attitudes, skills, and confidence in making healthy food and activity choices can have a significant impact on individual well-being [10]. The use of praise, inclusion, modeling and reinforcement from peers and mentors fosters safe and supportive relationships and increases the likelihood of youth embracing new behaviors [11, 12]. In addition to relationships and cultural aspects, programs that include “hands-on” activities are better able to keep children engaged, providing opportunities to gain new skills and confidence, and increasing lesson retention [13]. Interventions, therefore, should seek to create an inclusive, hands-on, and encouraging environment where children feel empowered to practice new healthy behaviors and connect these behaviors to their family. Yet, health promotions programs that emphasize solely intrapersonal change miss the influence of social and environmental contexts in which individual behaviors occur and are reinforced [14].

At the interpersonal and organizational levels, programs must consider family member support and involvement. Hispanic families place significant value on family structure and dynamics, which is hypothesized to be a protective factor for health outcomes, despite generally lower socioeconomic status [15]. Strong family cohesion positively impacts nutritional choices in youth, including increased breakfast and vegetable consumption and decreased soda intake [16, 17]. Family cohesion has also been linked to lower psychological distress [18], a greater self-reported well-being [19], and positive influence on youth physical activity [20]. Similarly, family cohesiveness positively predicts teenagers meeting the recommendations for adequate PA, a relationship mediated by increased self-esteem [21].

There is much to benefit from designing a Latinx nutrition and physical activity program that aims to strengthen family cohesiveness. Previous after-school physical activity and nutrition interventions have recognized this need to engage families for an effective program. Despite the desire to involve families heavily in interventions, few programs have been able to achieve high levels of consistent family participation [22, 23]. Rather than simply encouraging parental engagement, programs that successfully collaborate with families in the creation, implementation, and evaluation of youth programs lead to improved nutrition and physical activity measures among participants, and greater confidence among parents in their ability to encourage healthy behaviors in their children [24]. These methods help programs capitalize on strong social and family values to create inclusive, culturally based environments for students and families to thrive.

At the community and public policy level, systems and social norms can make it challenging for students and families to access and engage in health-promoting behaviors. When designing a Latinx community-based nutrition and physical activity program, it is necessary to address the specific health goals within the broader cultural contexts in which people live [25]. The value of community within social networks is demonstrated by social support being one of the strongest indicators of physical activity within the Hispanic community [8]. Peer support programs that allow children and families to share best practices from the intervention with peers, friends, and the greater community may effectively promote behavior change. Studies of peer intervention have demonstrated effectiveness at harnessing the power of a trained peer mentor to provide an intervention [26–28]. To provide the best chance for success, like other community-engaged programs [29–34], Latinx programs need to intersect across the ecological spectrum.

Evidence-based physical activity and nutrition programs may provide families with tools to improve their health if they are both culturally appropriate and community facing in their programming before, during, and after development. The SEM is the basis of the current analysis, which suggests that health behaviors result from influences at multiple levels: individual, interpersonal, organizational, and community [35]. However, no studies have investigated the interplay of synergistic factors that could make these types of community programs successful. We adapted the SEM to emphasize three main components of a physical activity and nutrition program with the Latinx community, Families Inspired Together 4 Youth Empowered to Succeed (FIT 4 YES), that we identify as major contributors to its success within individual families as well as the school community. 1) Opportunities for engagement. This included hands-on teaching strategies, the option to try multiple new activities, and assistance managing time and cost barriers for participants and families. 2) Strength in program relationships. FIT 4 YES leaders had been involved in the school community for years building trust through three prior iterations of this program, focusing on community needs in the development and providing constant encouragement for families throughout the program. 3) Interplay of common themes. All themes derived from family interviews after the program play a role in creating a space for students and families to grow together and improve their health and wellness.

## Methods

### Intervention

FIT 4 YES, a physical activity and nutrition program, began recruiting fourth and fifth-grade students in 2017 with active programming beginning in February 2018 from an urban Milwaukee middle school with a Hispanic population contributing to 95.8% of student enrollment [36]. FIT 4 YES was delivered 4 days per week after school plus adult times to ensure family participation. All programming was offered in both English and Spanish with a translator always available. Examples of evidence-based educational tools that were used in the sessions included USDA fresh fruit and vegetable program, Food and fun after school®, Team Nutrition, and Farm to Table combined with active learning including meal planning and preparation, “fast” meals, family FIT nights, fitness classes and workout partners for parents and active family weekend events. Students and families engaged in many outdoor activities they had never tried before including biking, camping, rock-climbing, cross-country skiing, downhill skiing and snowboarding, kayaking, canoeing, sailing, and hiking. Families continued these activities independently with program support by free use of shared equipment. FIT 4 YES dynamically evolved with feedback from our Community Advisory Board (three families, community advocate, and staff/key personnel) to ensure appropriate cultural programing and to involve the community continuously with the development of the program. Students who made up the cohort in the first year were invited to return in subsequent years. 38 students and their families were consistent throughout the program.

### Interviews

In the third year of the project, families were invited via email and face-to-face to participate in an in-person interview to discuss how the program impacted the children and families that participated. Interviews were conducted by author DN, PhD, male associate professor in the department of family and community medicine at the Medical College of Wisconsin. DN has advanced training in community-based participatory research in a variety of settings and understands how to work with community members and organizations. His mixed methods work based within communities have enabled a better understanding of treatment programs that are relevant to the communities in which he works. He led the qualitative analysis component of evaluation, specifically of behavioral changes occurring around family, food and PA. DN has worked extensively with Marquette University and the United Community Center (UCC) prior to study commencement. Participants were aware of the reasons for doing the research; all participants were given a letter of consent and were volunteers for the interview process.

Due to the COVID-19 pandemic, in-person interviews were moved online to a secure Zoom platform. Open-ended interviews were conducted in English and Spanish or both, depending on the family preference. If families spoke Spanish, an interpreter (MC) was present to translate the narrative between the family and researcher. All interviews were recorded and transcribed by a professional transcriptionist. Transcripts were not returned to participants for comment or correction. No repeat interviews were conducted. All participants voluntarily completed the interview process. Important characteristics included participation in the FIT 4 YES program, a parent present for the interview, willingness to speak of their experiences, and completion over Zoom or telephone due to the pandemic. Questions and guides were not provided ahead of time and were not pilot tested. Questions were approved by the native Spanish speaker for cultural sensitivity. The interview guide focused on the influence of the FIT 4 YES program on the child and family, and the aspects that participants may use in the future. Field notes and bracketed comments were made and talked about during the interview. Interviews lasted between 20 and 45 min.

At the start of the FIT 4 YES program, participants included fourth (*N* = 16) and fifth grade students (*N* = 22). From February 2018 through March 2020, 38 students were consistent with the program. After the program completed, the team conducted 24 interviews and Table 1 contains the participant demographics.

**Table 1:** Interview participant demographics. This table shows the breakdown of interview participants

**Interview participant demographics**	#
**Interview Total**	**24**
Mother	6
Mother and Student	15
Mother, Father, and Student(s)	2
Mother, Student, and Sibling	1

### Data analysis

We used a qualitative, grounded theory approach to analyze the data, which focuses on emerging themes from the interviews to develop theories. This process was similar to the one used by one of the investigators in previous studies [5, 31, 37–39].

Three medical student researchers joined the team after the interview process. Open coding and a constant comparison approach were used on an early transcription with the student researchers and the senior mentor to develop the initial coding structure [39]. The team conducted additional transcriptions to create the code book, consisting of the identified theme, the definition of the phenomenon, and examples of narrative text. After each coding, the students met with the senior researcher to discuss and rectify any individual differences. After four transcripts, no new major themes developed, representing thematic saturation. The team coded the remaining transcripts and met weekly to go over the results. Additional transcript coding gave rise to subthemes within each major theme and repeated phrases within the SEM and a new model emerged. Inter-coder reliability was not calculated, instead it was achieved with weekly discussions until agreement was met. Each transcript was read in totality by three medical students individually, with the senior researcher reviewing aspects of the interviews that were discussed for rectification purposes.

When approximately half the transcripts were coded, the entire team from both universities and the community partners gathered for a virtual presentation of the results and the progress made to date. Comments were recorded, questions were clarified and adjustments to phrases and position of the model were made. There was strong agreement between the partners as to the content and the way the model was envisioned. Participants were not invited to the initial debrief of findings due to the ongoing pandemic and the intention to create a follow-up study for feedback and reactions from parent participants.

## Results

Seven major themes emerged as recurring factors in each of the interview transcripts. These themes are consistent with the data presented and the findings.

### Family dynamics

Cohesion between family members and interactions providing support define Family Dynamics. Three subcategories further divide the category: the relationship between the parent and child, family involvement in the program, and family structure/routine. Throughout the interviews, participants explained that the program contributed to spending more time together as a family, time that was used to discuss topics ranging from healthy eating to coping with stress, creating deeper connection between family members. The extra time together was also spent enjoying outdoor activities, cooking, and grocery shopping, all as a family unit. Parents have even commented on the opportunity to learn from their children, participate with them in the program, and attend informational sessions on high blood pressure for example, causing one parent to go to the doctor and find out she has hypertension.

*"Making food together is helping us a lot at least to have a conversation about what they’re doing on the cooking days and where we do when we come to do something like a family. We’re able to talk."*

### Social dynamics

Social dynamics relate to the interactions between peers and mentors outside of the family. These dynamics included openness and encouragement among their peers. Multiple quotes from interviews describe the benefits of the social aspect of the FIT 4 YES program. Parents comment on their children becoming more outgoing, and students remember the friendliness of their peers and the relationships they created from participating in the program. Many interviews describe the encouraging environment of FIT 4 YES, and the importance of having role models for their children outside of the family structure for support.

*"I think definitely having those people there to support you through all those stages... It's always nice to have those people, these mentors that are available to you to talk to you or more than just having to, like I said, to talk to your parents because they know you on different levels or outside of school and to actually have those people there for you."*

### Independence

The category of independence relates to the gradual learning of self-sufficiency and responsibility. Parents comment on their children taking greater responsibility for themselves and their own health. Examples range from getting their backpacks ready before school, creating their own goals such as not drinking soda, and helping their parents with meal preparations. Many parents were proud to say they could trust their child to help cut vegetables for dinner and use the stove. It created another avenue for connection, moving from taking care of their child to encouraging their child’s growth and skill development.

*"I just see Alex being … more independent, and I think that’s helping him more to other like hobbies like he took up drawing recently... I think that’s what I like about it... growing basically."*

### Trying new and healthy things

FIT 4 YES provided support for the participants to learn about and participate in unfamiliar activities that promote health and wellness. This category spoke to the abundance of new activities that participants had the opportunity to experience within the program, some of which were able to be brought home and introduced to younger siblings. A few children were able to describe how exercising and stretching helped relieve stress they feel from school, ultimately improving their academic performance. The cooking classes from the program also introduced the participants to healthier recipes and provided them with hands-on opportunities to learn how to plan and prepare meals at home. Parents and students both commented on the increased variety of fruits and vegetables offered that participants were willing to try.

*"Shes been more open to different things so it’s not just one specific sport now that she’s interested… now she’s more open and she’s been asking me, hey, can I join this? I wanna try this out...’It's opening up her perspectives to all different types of things to do to stay active."*

### Changes in routine

As an extracurricular activity, FIT 4 YES provided a deviation from the typical daily schedule with a chance to engage students in healthy behaviors after their school day. Students and parents alike commented on the benefits of being active in the program rather than sitting on the computer playing video games after school. The program provided space for the participants to explore new hobbies that extend beyond the program into their routines at home. For example, one student commented on the extra walks she now regularly takes with her father, and another parent described her daughters newfound love of swimming.

*"I like that after school we get to, instead of sitting down, we get to have fun by exercising."*

### Confidence

Confidence is defined as feeling self-assured in one’s own abilities, interests, and qualities. Repeatedly, the students described feeling more comfortable with themselves and noticed improvements in their own shyness and social skills. Parents stated feeling proud of their children, watching them try new activities and dive deeper into exploring their own personalities.

*"It helped me be more like comfortable with like who I am… Like at first, I didn’t like to do stuff and I was shy. I’m still shy but not as much as I was when I first joined."*

### Safety

The feeling of security in an environment and belief that one is protected from judgment by the people around them defines the category of safety. In many of the interviews, student participants described the supportive environment created by both the program and the other students. Parents commented on the comfort they had knowing their child could go to trusted adults if there was ever a problem.

*"I feel like [FIT 4 YES is] a safe space for more people. Like there’s not much fighting or like bullying there. It’s just where we can all have fun and get together and learn."*

## Discussion

Never in time has there been a more significant movement of people from one country to another. Across the U.S. and world, there is a need for programming that supports the health and well-being of people that move from one part of the world to settle in another. Programs developed by academic institutions may either gloss over or omit cultural components, missing an opportunity not only to connect with families but also to deepen the connection between culture and health [8]. Developing culturally appropriate programs emphasizing strong cultural values such as family and respect has shown to deepen child relationships, engage family members in health-related change, and improve the family health status [40]. Using the SEM as a framework, we sought to identify factors within FIT 4 YES that supported youth capacity to learn and achieve fitness levels that can be applied in both the present and future. In addition, the ideas and ideals adopted by the participants were brought home to the families and often incorporated into the family structure. The components of the present project fit well with the SEM.

**Fig. 1:**
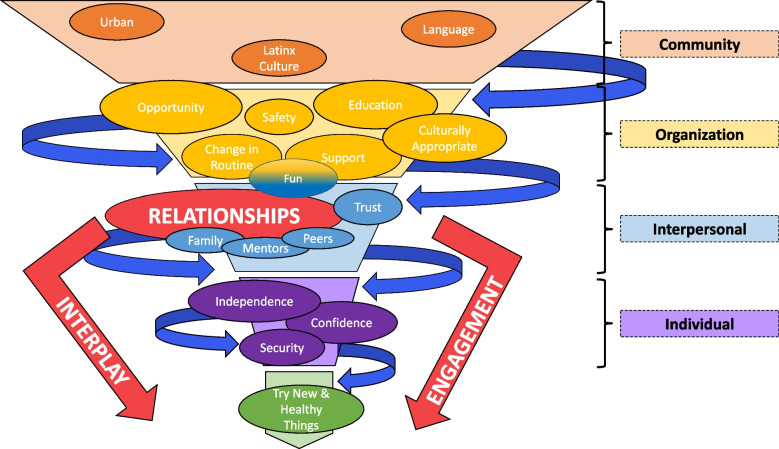
“It’s about being healthy:” Community Based Health Promotion Model. The creation of this novel model is based on themes that emerged from family interviews that allowed for program success. Current iterations of the social ecological model (SEM) demonstrate the effects of multi-level interventions on the individual; however, the static nature of these models fail to represent the synergy and movement many of these effects have with each other. Our model integrates the levels of the SEM with aspects of the FIT 4 YES program that were necessary to empower participants to engage with healthy behaviors. Each of the factors included are linked in such a way to create a funnel effect down to the individual level allowing for change within the larger community

The use of the SEM is not new within the literature [2, 7]. However, the dynamics of community engagement and the effectiveness of programs across the model often do not come through. Although consisting of interconnected circles, the SEM appears static. Effective programs are anything but static and there is a need for new models and concepts to emerge. The richness of the data from family interviews developed into such a new and dynamic model over time. Early versions consisted of interconnected and adjacent circles similar to a model developed by Waniger [4] in describing the process of caregiving. This model, too, did not seem to capture the movement that was occurring as an expression of the phenomenon. Over time, the model emerged into a vortex with activity occurring between the components. The most recent edition is presented in Fig. 1. We would like to discuss some of those components and the implications for using this model in future programs and research.

The seven defined aspects for success of the FIT 4 YES program can be more broadly categorized into three “ingredients” to make a community-facing nutrition and physical activity program successful:

### Strength in relationships

Strength of relationship is a key component of the model and observed in all aspects of the program. These essential relationships included those between participants and program leaders, peers, and family members. This program created a space for both children and families to deepen multiple relationships. Because the program leaders had been previously involved with the community, they built deeper connections with the children and encouraged their families to engage with different aspects of the program using their preferred communication styles. This level of trust allowed for the relationships to extend beyond the confines of the program, as participants brought home knowledge and skills and continued to develop and share this excitement with those around them.

Not only were these connections brought home, but the participants created their own environment of safety and peer support. Adolescence is a challenging time for youth as individuals seek to fit in and tend to be body conscious. Children at this stage of development are often both the subject and object of bullying, teasing, and making fun of one another. Yet, in this environment, there was little of that happening. During one of the observation sessions, a group playing frisbee displayed this dynamic. When a young man missed a catch and was struck on the cheek with the frisbee, several of the other children gathered around him to offer support and encouragement instead of teasing him. Once he composed himself, he was brought back into the game with open arms. Examples like these were witnessed regularly during physical activity and the nutrition sessions focused on cooking. One family commented extensively on the “safe space” that FIT 4 YES provided:

*Son: “I feel like [FIT 4 YES is] a safe space for more people. Like there’s not much fighting or like bullying there. It’s just where we can all have fun and get together and learn.”*

*Mother: “This program is safe and if they ever need to talk to one of the adults, they have that opportunity to talk to them and for them to feel safe.”*

*Son: “It felt good. I could tell that I wasn’t alone and that I had people with me to go.”*

*Mother: “I think it’s awesome that other students in the program are there for each other and are helping one another out and I think that’s what it should be about.”*

### Engagement

The compact nature of urban environments can be a challenge for healthy eating and active living. The UCC is situated on the near south side of Milwaukee, the densest living conditions in Milwaukee. Constant traffic and safety concerns make for a challenging environment for play. In addition, the one large chain grocery store available to residents has since moved, leaving only bodega-style convenience stores and the largest grocery store nearly two miles away. FIT 4 YES, situated within the school and adjacent to the UCC, used the school’s resources and a neighboring park and was a safe place for children and families to get involved in healthy eating and fitness activities.

FIT 4 YES employed hands-on engagement strategies for both the nutrition and physical activity components of the program. The emphasis on fun allowed students the space to try new foods and practice components of the lesson with less pressure, and to work towards bringing home knowledge and skills for future use. One example of skill development and fun engagement was working on knife skills – learning to use sharp kitchen knives to cut vegetables. Each participant took the instruction seriously, only picking up their knives during a knife demonstration when the instructor told them to do so. Even parents noticed the skills that the children developed. Some commented that there was a seeming maturity in the children following the program. Others commented that their children could now make a meal using knives, stoves, and other utensils and not “burn down the house” said with a smile.

Interviews frequently commented on how the FIT 4 YES program engaged the family as well as the students. This led to strengthened family relationships and changed family routines. Parents were grateful for their opportunity to learn with and spend more time with their children through their inclusion in programming, and often remarked on the programs ability to give their children experiences they felt unable to provide themselves, such as skiing, by providing transportation and free use of shared equipment. Students brought home lessons and skills they learned from the program to share with their parents and siblings and became more aware of the food they were eating at home. While grocery shopping with mom or dad, they initiated looking at food labels and encouraged their parents to purchase healthier options. FIT 4 YES allowed families to connect more outside of the program as children wanted to discuss what they were learning with their parents and practice their new skills with them. Routines and habits also changed as families spent quality time by playing outside, walking, or cooking together, rather than sitting and watching TV.

*“I think, you know, Hispanics…it’s hard for us to do change I think overall in any person. Change is hard but I am thankful for all the events, for all the classes, for everything the forum has offered. I feel that the classes, the retreat, any event that they have involved with the family and the kids have made me feel closer to my child and I have learned a lot from him and from the school and from the program, and I am forever going to be thankful for everything. So, I do appreciate all the help you guys offer.”*

### Interplay of all ingredients

The themes that emerged from the family interviews (family dynamics, social dynamics, independence, trying new and healthy things, changes in routine, confidence, and safety) led to the creation of our novel model that we believe allowed for program success. Current iterations of the SEM demonstrate the effects of multi-level interventions on the individual; however, the static nature of these models fail to represent the synergy and movement many of these effects have with each other. Our model integrates the levels of the SEM with aspects of the FIT 4 YES program that were necessary to empower participants to engage with healthy behaviors. Each of the factors included are linked in such a way to create a funnel effect down to the individual level allowing for individuals to try new things.

Beyond the interplay of the components is the movement that occurs between the components. Where there was once a model that showed a downward flow of components, the final version showed a swirling series of arrows that moved through and down the model. This model illustrates that none of these themes can stand alone in a program. Each aspect contributes to the strength in relationships, engagement, and interplay, allowing for the creation of a community-focused health promotion program, and allows the flexibility to emphasize the themes that may work best for the specific community.

*“I think it has helped him just like how to interact with other people, like with the cooking and with the sports like sharing, helping each other, encouraging each other…giving positive comments to each other…I like the community that they’ve built together”*

## Conclusions

The three larger “ingredients” (strength in relationships, engagement, and interplay) are aspects of community-centered health promotion programs that each have vital aspects when standing alone. When present and in play with each other, these components allow for the greatest impact within the family. We saw the creation of a community outside the program space that further integrated the knowledge, skills, and support beyond the individual level.

We discovered that the student participants in FIT 4 YES acted as catalysts for change for their families, leading to the potential for change within the larger community. Parents reported an increase in their children’s self-directed healthy behavior, encouraging examination of nutrition labels in the grocery stores, and helping with dinner preparation using knowledge and skills gained in the program. Students would inspire their siblings and friends to spend more time outside, increasing their physical activity through games and additional resources. By children encouraging their parents to attend the program, they not only helped strengthen the cohesion within their own families, but also between families and the community, creating a network with emphasis on supporting and learning with each other, rather than trying to change alone.

Commitment to strengthening family cohesion through a health-promotion program provides an opportunity to move from the individual to community level. Instead of focusing on the individual take-aways, programs can take it a step further to look at how family relationships are impacted, and how that may be leveraged for a larger community impact.

### Limitations

Qualitative research are not generalizable to another group, though it is believed that the number and depth of the interviews provides points that could be relevant to other groups. In addition, no outcome measures were completed. For this project, the emphasis was on the experience of the participants and their families, and the development of the model.

#### Public health implications

Something is missing from the current version of the SEM and does not quite express the level of synergy when creating a community-focused health promotion program. We created a novel model displaying this dynamic relationship based on family interviews that we believe can be utilized to support strong family cohesion and ultimately have a larger community impact. In the development of future programs, this model can be used to highlight the vital aspects necessary to create an environment promoting healthy changes on a community level.

The team views the model as a beginning point rather than an end. The limited number of models for wellness and nutrition means that with the development of this model, the next step will be to test the model to determine what components will stay and what may shift. Additional evaluation could also focus on the impact and reaction of the model by conducting additional interviews. In future interventions, the implementation of a toolkit could emphasize connections with the community by trained parent navigators. Finally, like many programs focused on wellness, there is a lack of long-term information on aspects of the project that stick with individuals and families over the long term. For now, this model demonstrates what might be possible when a robust program is developed with the community in mind.

## Supplementary Information

**Additional file 1.** Interview question guide. All questions were approved by a native Spanish speaker for cultural sensitivity.

**Additional file 2.** COREQ checklist. In accordance with BMC Series editorial policies, our manuscript reporting adheres to COREQ guidelines. 

**Additional file 3.** Participants’ Responses to Program. This table illustrates specific quotes from the participant interviews that best demonstrate each respective theme that emerged from the qualitative analysis.

**Additional file 4.** Prior Model Iterations. This figure demonstrates the evolution of our model and the shifts in the development from a simple flowchart to Venn-diagrams, to panels that illustrate the movement observed between and within each level.

## Data Availability

The data that support the findings of this study are available from the corresponding author upon request.

## Abbreviations

PA: Physical activity
FIT 4 YES: Families Inspired Together 4 Youth Empowered to Succeed
SEM: Social-ecological model
UCC: United Community Center
