# Supplementary material for: “It’s about being healthy”; a novel approach to the socio-ecological model using family perspectives within the Latinx community
Source: BMC Public Health. 2023 Jan 11;23:86. doi: 10.1186/s12889-023-15005-2 (PMC9833868; doi:10.1186/s12889-023-15005-2)
Supplement: Supplementary file 1 — Additional file 1. Interview question guide. All questions were approved by a native Spanish speaker for cultural sensitivity. [file 12889_2023_15005_MOESM1_ESM.pdf]

Additional file 1: Interview question guide. All questions were approved by a native Spanish speaker for cultural sensitivity.

Fit Families Questions

To parent:

Tell me about your son/daughter?

Can you tell me what it has meant to have your children participate in the Fit Families Program?

What have you noticed about your son/daughter?

How has this program impacted the family?

To child <u>or</u> to parent if child is not present

What might you remember from this program as you grow older?

To Both:

Is there anything else
