# Supplementary material for: “It’s about being healthy”; a novel approach to the socio-ecological model using family perspectives within the Latinx community
Source: BMC Public Health. 2023 Jan 11;23:86. doi: 10.1186/s12889-023-15005-2 (PMC9833868; doi:10.1186/s12889-023-15005-2)
Supplement: Supplementary file 3 — Additional file 3. Participants’ Responses to Program. This table illustrates specific quotes from the participant interviews that best demonstrate each respective theme that emerged from the qualitative analysis. [file 12889_2023_15005_MOESM3_ESM.pdf]

Additional file 3: Participants' Responses to Program. This table illustrates specific quotes from the participant interviews that best demonstrate each respective theme that emerged from the qualitative analysis.

Family Dynamics	Social Dynamics	Independence
"the nutrition aspect with the food, making food and all that together is helping us a lot at least to have a conversation about what they're doing here on the cooking days and where we do when we come to do something like a family. We're able to talk"	"I think it has helped him just like how to interact with other people, like with the cooking and with the sports like sharing, helping each other, encouraging each other... giving positive comments to each other... I like the community that they've built together"	"I just see Alex like being on his, you know, independent I guess, more independent, and I think that's helping him more to other like hobbies like he took up drawing recently... I think that's what I like about it... growing basically"
Since the program started we have been more active...we'll go for walks in the park... there is engagement from the family, you know, so we can keep the kids active as well"	"... getting him a little out of his comfort zone... sometimes he wouldn't try anything and just like be off in the benches or just sit there and I feel like now he at least sees other kids doing it or he sees their struggles and then hes willing to try it now... Seeing that not every kid is gonna be able to ride a bike or do something that he wasn't doing either at his age, I think helped him be more comfortable around people or also encouragement probably, you know from Ms. Kelly."	"Even when we go to the grocery store, he'll be checking that things don't have much sugars or he'll be checking everything like labels to see if they are healthyand everything. So I'm thinking it's gonna make him a lot better decisions with his eating. "
"I don't know if we're gonna have time... we'll make it work... it just flowed so naturally having him participate in activities and us just kind of being there with him or being part of it. It wasn't as hard as I thought it was gonna be. And it helped a lot that we could be part of those activities but it was just helping out with the cooking or cheering, helping the staff in any way that we could or just socializing with other parents as we were waiting for them to finish. So it really is really doable... We like it alot"	"It makes a difference for those people to actually wanna care and to actually wanna influence and make adifference in childrens lives. I wanna say thank you so much for being here this last 2 years and for helping her. For spending that extra time with her, you know, and being those role models that she needs outside of the house when I'm not there. Thank you so much"	**Safety**
"it's all about seeing what they're excited about and encourage them, let them know that even if they burn something or do something like that, it's okay. Try again. If you need my help, I'm here to help guide you. If I don't know, maybe we can learn together"	"I think definitely having those people there to support you through all those stages... its always nice to have those people, these mentors that are available to you to talk to you or more than just having to, like I said, to talk to your parents because they know you on different levels or outside of school and to actually have those people there for you"	"I feel like [Fit is] a safe space for more people. Like theres not much fighting or like bullying there. It's just where we can all have fun and get together and learn"
**Trying New and Healthy Things**	**Changes in Routine**	**Confidence**
"It helped me to try new fruits and vegetables... learned that like, what they have on the boxes, like labels. How much fat, calories, and all that and how many servings we should have... Like they taught us how to cook foods, like different stuff to make it healthier"	"I like that after school we get to like instead of sitting down we get to have fun by exercising... I like to be active,not just sitting down and doing nothing"	"I feel more confident [in the choices that I make]... And like I feel like I make smarter choices now"
"Shes been more open to different things so its not just one specific sport now that shes interested... now shes like more open and shes been asking me, hey, can I join this? I wanna try this out... its opening up her perspectives toall different types of things to do to stay active."		"It helped me be more like comfortable with like who I am...Like at first I didn't like to do stuff and I was like shy. I'm still shy but not as much as I was when I first joined."
