# Supplementary material for: “It’s about being healthy”; a novel approach to the socio-ecological model using family perspectives within the Latinx community
Source: BMC Public Health. 2023 Jan 11;23:86. doi: 10.1186/s12889-023-15005-2 (PMC9833868; doi:10.1186/s12889-023-15005-2)

Additional file 4: Prior Model Iterations. This figure demonstrates the evolution of our model and the shifts in the development from a simple flowchart to Venn-diagrams, to panels that illustrate the movement observed between and within each level.

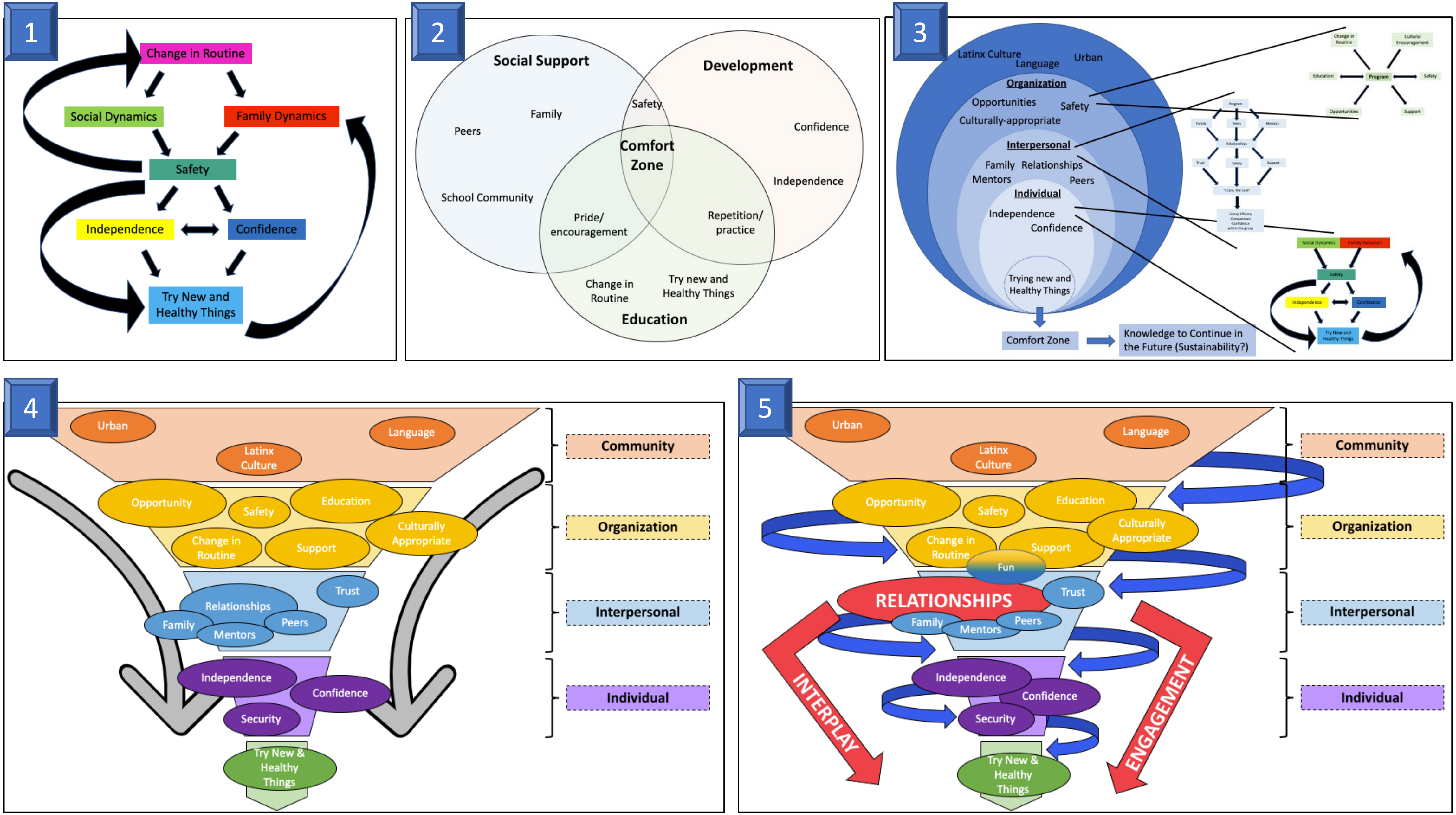

Supplement: Supplementary file 4 — Additional file 4. Prior Model Iterations. This figure demonstrates the evolution of our model and the shifts in the development from a simple flowchart to Venn-diagrams, to panels that illustrate the movement observed between and within each level. [file 12889_2023_15005_MOESM4_ESM.pdf]
